# Dynamics of neuromodulatory feedback determines frequency modulation in respiratory network

**DOI:** 10.1186/1471-2202-13-S1-P175

**Published:** 2012-07-16

**Authors:** Natalia Toporikova, Robert J Butera

**Affiliations:** 1 Laboratory for Neuroengineering, Georgia Institute of Technology, Atlanta, GA, 30332-0250, USA; 2 School of Electrical and Computer Engineering, Georgia Institute of Technology, Atlanta, GA, 30332-0250, USA

## 

Neuromodulators, such as amines and neuropeptides, alter the activity of neurons and neuronal networks. In this work, we investigate how neuromodulators which activate Gq-protein second messenger systems can modulate the frequency of bursting neurons in a critical portion of the respiratory neural network, the pre-Bötzinger complex (pBC). These neurons are a vital part of the ponto-medullary neuronal network, which generates a stable respiratory rhythm, whose frequency is modulated by neuromodulator release from nearby Raphe nucleus. Using a simulated 50-cell network of excitatory connected pBC neurons with a heterogeneous distribution of persistent sodium conductance and ER Ca2+, we determined conditions for frequency modulation in such network by simulating interaction between Raphe and pBC nuclei. We found that the positive feedback between the Raphe excitability and pBC activity induces frequency modulation in the pBC neurons. In addition, the frequency of the respiratory rhythm can be modulated via phasic release of excitatory neuromodulators from the Raphe nucleus. We further predict that the application of a Gq antagonist will eliminate this frequency modulation by Raphe and keep the network frequency constant and low. In contrast, application of a Gq agonist will result in a high frequency for all levels of Raphe stimulation. Our modeling results also suggest that high [K+] requirement in respiratory brain slice experiments may serve as a compensatory mechanism for low neuromodulatory tone.

